# Stability of Drinking Water Distribution Systems and Control of Disinfection By-Products

**DOI:** 10.3390/toxics11070606

**Published:** 2023-07-12

**Authors:** Qingwei Zhou, Zhengfu Bian, Dejun Yang, Li Fu

**Affiliations:** 1 School of Environment Science and Spatial Informatics, China University of Mining and Technology, Xuzhou 221116, China; yangdj81@163.com; 2 College of Materials and Environmental Engineering, Hangzhou Dianzi University, Hangzhou 310018, China; fuli@hdu.edu.cn

**Keywords:** drinking water distribution systems, water quality, corrosion products, biofilm interactions, disinfection by-products

## Abstract

The stability of drinking water distribution systems and the management of disinfection by-products are critical to ensuring public health safety. In this paper, the interrelationships between corrosion products in the network, microbes, and drinking water quality are elucidated. This review also discusses the mechanisms through which corrosive by-products from the piping network influence the decay of disinfectants and the formation of harmful disinfection by-products. Factors such as copper corrosion by-products, CuO, Cu_2_O, and Cu^2+^ play a significant role in accelerating disinfectant decay and catalyzing the production of by-products. Biofilms on pipe walls react with residual chlorine, leading to the formation of disinfection by-products (DBPs) that also amplify health risks. Finally, this paper finally highlights the potential of peroxymonosulfate (PMS), an industrial oxidant, as a disinfectant that can reduce DBP formation, while acknowledging the risks associated with its corrosive nature. Overall, the impact of the corrosive by-products of pipe scale and microbial communities on water quality in pipe networks is discussed, and recommendations for removing DBPs are presented.

## 1. Introduction

The quality of potable water is intimately intertwined with human well-being and health. As China living standards ascend, the expectations for drinking water’s purity have correspondingly heightened. Offering safe, superior-quality drinking water carries significant strategic implications in enhancing people’s quality of life, fostering societal harmony, and safeguarding national security. Historically, the primary focus has been placed on the preservation of water sources, the innovation of contemporary water treatment technologies, and the revolution of drinking water treatment processes, with the final link in the supply chain–the water distribution network–often overlooked [1]. Generally speaking, the various quality indicators of treated water can achieve national drinking water standards [2]. However, drinking water must traverse long distances from treatment facilities to end-users, engaging in complex physical, chemical, and biological reactions as it contacts the inner walls of the network and surfaces of attached equipment, resulting in changes to water quality. This can lead to instances of water turbidity, coloration, elevated metal content, and bacterial exceedances of the drinking water standard at user endpoints, engendering “secondary contamination” of the network [3].

Statistics show that the qualification rate of treated water in China declines by nearly 20% after traversing the water supply network and secondary water supply equipment [4]. Survey analysis of 45 domestic cities reveals that network water turbidity increased by 0.38 NTU compared to treated water, color increased by 0.45 CU, iron concentration increased by 0.04 mg/L, manganese concentration increased by 0.02 mg/L, total bacterial count increased by 18 CFU/L, and *Escherichia coli* increased by 0.4 per/L [5]. As can be seen, preventing the deterioration of network drinking water quality and safely transporting high-quality drinking water to end-users are pressing issues that the water supply industry needs to address.

The mechanism of water quality deterioration within the network is complex. However, it can be affirmed that the corrosion of the network directly and indirectly affects the safe transmission of the water supply network [6]. Corrosion in the network creates nodules that provide important habitats for microbial growth, reducing the effect of disinfectants and increasing microbial safety risks [7,8]. The corrosion products of the network, microbes, and drinking water quality interact and influence each other [7]. Network corrosion products alter the progress and state of chemical reactions in the network through oxidation or catalytic effects, thereby changing the quality of the water. The change in water quality will affect the stability of scale and the microbial metabolism. The microbial metabolism can, in turn, affect the structural composition of network corrosion products, and its metabolites can directly impact the quality of the water.

## 2. Stability of Water Quality in the Network

The stability of water quality within the supply network comprises both chemical stability and biological stability [9]. Chemical stability refers to the behavior of inorganic element migration and transformation within the network, primarily including sediment scaling and pipeline corrosion; biological stability refers to the problem of microbial regrowth in the network [10]. The rough or protruding inner surfaces of the water supply network system provide effective microenvironments for microbial growth, while biofilm growth often accelerates pipe corrosion [11].

### 2.1. Chemical Stability of Network Water Quality

Corrosion in the network is defined as the process wherein metal ions are released into the water or form scale on the pipe wall during metal oxidation. Network corrosion can be divided into general corrosion and localized corrosion. General corrosion forms a relatively uniform corrosion scale, while localized corrosion forms lumpy corrosion nodules [12]. Corrosion products are composite bodies formed by the combination of rust (metal oxides) and sediments (precipitates and colloidal particles from the water). Different pipe materials produce different corrosion products when they corrode [13,14]. In cast iron pipes, iron oxides are the primary corrosion products [15,16]; in copper pipes, copper oxides are the primary corrosion products; in lead pipes, lead oxides are the primary corrosion products [17,18]. In the water supply network in China, cast iron pipes are most widely used. Therefore, this study uses cast iron pipes as an example to introduce the formation of the scale layer.

**Figure 1:**
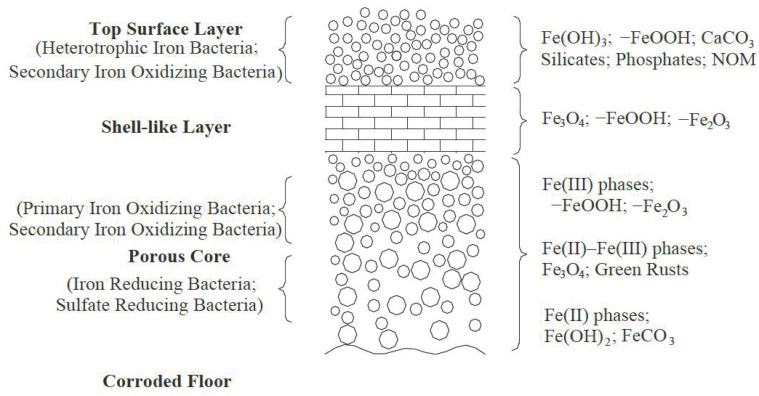
Structural model of corrosion nodules in the network currently. The main mechanisms of cast iron corrosion include the Siderite model and Lytle’s Iron–Sulfur transformation model [13,19,20].

As shown in Figure 1, the nodular scale has a three-layer structure, which consists of a surface deposition layer, a dense hard shell layer, and an inner porous loose layer [13,19]. The corrosion products in the cast iron pipe scale layer mainly include magnetite (Fe_3_O_4_), hematite (α-Fe_2_O_3_), maghemite (γ-Fe_2_O_3_), goethite (α-FeOOH), akaganeite (β-FeOOH), lepidocrocite (γ-FeOOH), siderite (FeCO_3_), ferric hydroxide (Fe(OH)_3_), ferrous hydroxide (Fe(OH)_2_), ferrihydrite (5Fe_2_O_3_·9H_2_O), calcite (CaCO_3_), magnesium carbonate (MgCO_3_), and green rust [13,20]. Fe_3_O_4_ and α-FeOOH are usually the main iron oxides in corrosion scale. Fe_3_O_4_ contains divalent iron, which is conducive to electron transfer and has good conductivity; α-FeOOH only contains trivalent iron, is not conductive, and is therefore very stable. β-FeOOH and γ-FeOOH are homologous forms of α-FeOOH, formed in the early stages of corrosion and eventually transformed into Fe_3_O_4_. Under aerobic conditions, α-FeOOH, β-FeOOH, and γ-FeOOH react with Fe(OH)_2_ to form Fe_3_O_4_, with the order of preference being: β-FeOOH > γ-FeOOH >> α-FeOOH [21]. Therefore, α-FeOOH and Fe_3_O_4_ are relatively stable corrosion products.

The Siderite model [22]: The corrosion of the iron matrix and dissolution of divalent iron components are the main reasons for the increase in iron concentration in the water phase. In bodies of water with strong buffering capacity, Fe^2+^ first reacts with CO_3_^2−^ to form FeCO_3_, which is then slowly oxidized to form a robust protective layer on the iron base, thus inhibiting the corrosion of the iron matrix.

The initial step:

(1) $$\mathrm{Fe}\to {\;\mathrm{Fe}}^{2+}{+2e}^{-}$$

(2) $$0.5O_{2}{\;+\;H}_{2}{O+2e}^{-}\to \;{2\mathrm{OH}}^{-}$$

(3) $${\mathrm{HCO}}_{3}{}^{-}{+\mathrm{OH}}^{-}\to {\;\mathrm{CO}}_{3}{{}^{2}}^{-}{+H}_{2}O$$

Step 2:

(4) $${\mathrm{Ca}}^{2+}+{\mathrm{CO}}_{3}^{2-}\to {\;\mathrm{CaCO}}_{3}$$

(5) $${\mathrm{Fe}}^{2+}{+\mathrm{CO}}_{3}^{2-}\to {\;\mathrm{FeCO}}_{3}$$

(6) $${2\mathrm{Fe}}^{2+}+0.5O_{2}+4{\mathrm{OH}}^{-}\to \;2\alpha -\mathrm{FeOOH}\left(s\right)+{\;H}_{2}O$$

Step 3:

(7) $${2\mathrm{FeCO}}_{3}\left(s\right)+0.5O_{2}+{\;H}_{2}O\to \;2\alpha -\mathrm{FeOOH}\left(s\right)+2{\mathrm{CO}}_{2}$$

(8) $${3\mathrm{FeCO}}_{3}\left(s\right)+0.5O_{2}\to {\;\mathrm{Fe}}_{3}O_{4}\left(s\right)+3{\mathrm{CO}}_{2}$$

Lytle’s Iron–Sulfur Transformation Model [8]: In the anaerobic environment of the porous, loose layer inside the corrosive scale, sulfate-reducing bacteria utilize organic matter and hydrogen as energy sources, reducing the sulfate, which acts as an electron acceptor, to sulfide.

(9) $$\mathrm{Fe}\to {\;\mathrm{Fe}}^{2+}+{2e}^{-}$$

(10) $${\mathrm{SO}}_{4}{{}^{2}}^{-}{+\;4H}_{2}\to {\;S}^{2-}+{4H}_{2}O$$

(11) $${\mathrm{Fe}}^{2+}{+\;S}^{2-}\to \;\mathrm{FeS}$$

Sulfide, moreover, can reduce FeOOH, releasing Fe^2+^ into the water.

(12) $$2\mathrm{FeOOH}\left(s\right)+{\;H}_{2}S\;+{\;4H}^{+}\to {2\mathrm{Fe}}^{2+}{+\;S}^{0}{+\;4H}_{2}O$$

(13) $$5\mathrm{FeOOH}(s)+{\;H}_{2}{S+8H}^{+}\to {5\mathrm{Fe}}^{2+}{+\mathrm{SO}}_{4}{}^{2-}{+\;6H}_{2}O$$

**Figure 2:**
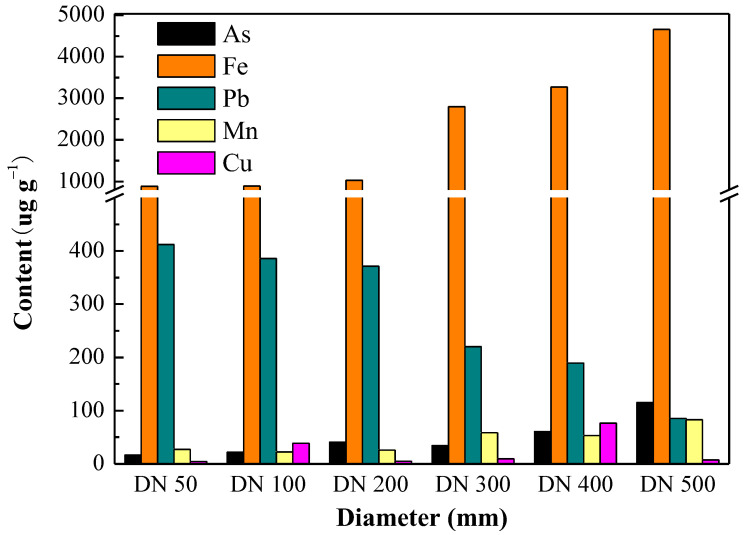
Elementary composition of corrosion layer in cast iron pipes [23].

However, the formation process of the corrosion layer in the water network is still subject to further research. Concurrently, there are a small amount of other metal elements in cast iron pipes, such as manganese (Mn) and copper (Cu). Trace amounts of metal elements are also present in factory drinking water. These metal elements precipitate onto or are adsorbed onto the corrosion layer (Figure 2), forming other corrosion products, such as MnO_2_ and CuO, through a series of oxidation-reduction reactions [23].

Primary factors influencing water quality in relation to corrosion encompass pH, alkalinity, inorganic ions, dissolved oxygen, and organic matter, amongst others.

pH: Within the potable water pH range (6.0–9.0), iron corrosion products generally deposit in a solid form on the interior pipe wall surfaces or are directly discharged into the water in the form of ferrous iron. According to primary battery corrosion principles, an increase in pH inhibits iron corrosion. However, certain studies have discovered an increased pH exacerbates iron corrosion, resulting in uneven corrosion, which prompts the formation of nodular protrusions [24]. Furthermore, some research found a pH decrease beneficial in inhibiting the release of iron corrosion products [25].

Alkalinity: An increase in alkalinity can inhibit iron corrosion [26,27]. On one hand, CO_3_^2−^ reacts with Ca^2+^ and Mg^2+^ to form insoluble CaCO_3_ and MgCO_3_ precipitates, which deposit on pipe wall surfaces, preventing the iron base from contacting dissolved oxygen, hence inhibiting iron corrosion. On the other hand, increasing CO_3_^2−^ concentration aids in generating FeCO_3_, which has a lower solubility than Fe(OH)_2_, ultimately forming a uniform, dense corrosion layer through a slow oxidation process, thus protecting the iron base.

Inorganic anions: SO_4_^2−^ and Cl^−^ are the primary anions forming dissolved salts in water. High concentrations of SO_4_^2−^ and Cl^−^ accelerate the iron corrosion and release processes. Their increased concentrations enhance the water’s conductivity, promoting primary battery corrosion reactions. Additionally, they form complexes with the Fe^2+^ produced during corrosion, facilitating iron release.

Dissolved Oxygen: Dissolved Oxygen (DO) is the primary electron acceptor in iron corrosion reactions. Increased DO concentration accelerates primary battery corrosion reactions. However, iron corrosion can still occur under anoxic conditions [28]. Moreover, DO can oxidize the Fe^2+^ generated from corrosion into ferric iron (Fe(III)), thus inhibiting iron release. Consequently, DO plays a complex role in iron corrosion and release processes, exhibiting different functions under different conditions.

Organic matter: Large organic molecules can reduce iron corrosion rates by accumulating and coating the pipe network surfaces over time. The combination of organic and inorganic matter also assists in forming a stronger protective layer [29]. Yet, organic matter can alter the redox potential of the reaction system, thereby increasing the concentration of soluble ferrous iron in water. Additionally, organic matter can form soluble complexes with different iron forms, promoting iron release [30].

When switching water sources in the pipe network, differences in water quality between the old and new sources can easily disrupt the network’s equilibrium, leading to water quality degradation. Widespread degradation of end-user water quality due to water source changes has been reported multiple times domestically and internationally. Cities such as Tucson, Southern California regions, and Tampa, Florida, all experienced severe “yellow water” phenomena after their water supply source was switched from groundwater to surface water [13]. In 2008, when raw water from Hebei Reservoir reached the Beijing tap water network via the South-to-North Water Transfer Project pipeline, “yellow water” appeared in certain areas. These instances indicate the significant impact water quality has on the stability of pipe network scales. In order to control the scaling and corrosive tendencies of water quality, scholars have established stability indices for water quality. These indices are divided into two major categories: those based on CaCO_3_ dissolution equilibrium, such as the Langelier Saturation Index, Ryznar Stability Index, Calcium Carbonate Precipitation Potential, and Aggressive Index; and those founded on other water quality parameters, such as the Larson Index [31]. In a previous study, Luo et al. report that the yellow water phenomenon often appears when desalinated seawater entered the old ferruginous water supply systems [32]. According to the Standards for Drinking Water Quality (GB5749-2006), the pH of this desalinated seawater is 8.09, which generally meets the requirements (6.5–8.5). So, pH and alkalinity are selected as quality control indices for desalinated seawater quality chemical stability. Considering the impact of the electrochemical reaction balance and colloid in water, this paper also suggests that Langelier Saturation Index and Ryznar Index shall be introduced to indicate the pipe water quality chemical stability [32]. Therefore, pH, alkalinity, Langelier Saturation Index, and Ryznar Index are selected to be the quality control indices of desalinated seawater quality chemical stability. The aforementioned example demonstrates that as pipe network scales and source water quality vary across regions, preventative measures and emergency responses necessitate further site-specific investigations.

### 2.2. Biological Stability of Pipe Network Water Quality

**Figure 3:**
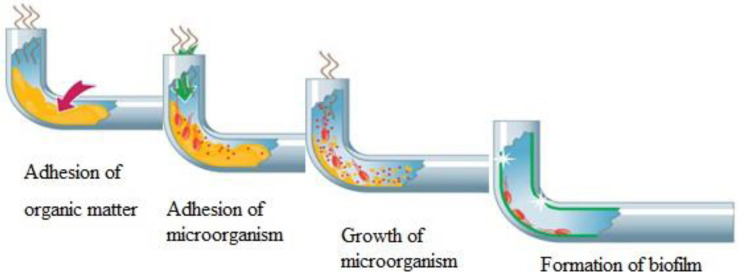
The schematic of biofilm formation [11].

The formation of biofilms on the interior walls of water supply networks is an intricate, dynamic process (Figure 3), primarily encompassing five stages: substrate surface property regulation; reversible microbial adhesion; irreversible microbial adhesion; formation of surface micro-communities; and appearance, detachment, and proliferation of biofilms [11]. The attachment, growth, and stability of biofilms on the inner surfaces of networks are incremental, influenced by an array of factors such as pipe material, water quality indices, and hydraulic conditions.

During the substrate surface property regulation stage, diverse organic substances in the water (e.g., proteins and polysaccharides) are adsorbed onto the interior surface via hydrophobic interactions and surface chemical reactions upon contact between the network and water [33]. The adsorbed organic matter negligibly impacts the roughness of the surface and its heat transfer characteristics, yet it can alter the surface charge of the pipe material while providing the necessary nutrients for the growth of bacteria and other microbes, thus fostering favorable conditions for microbial adhesion [34].

In the reversible microbial adhesion stage, microbes in the network water, under the influence of van der Waals forces, electrostatic forces, and hydrophobic interactions, make contact with the pipe wall and subsequently adhere to it. However, the adhered microbes are prone to detachment under the force of water flow, resulting in a state of unstable reversible adhesion.

During the irreversible microbial adhesion stage, microbes excrete copious amounts of extracellular polymeric substances (EPS), which possess adhesive properties, following reversible adhesion [35]. EPS firmly bonds the microbes and the interior wall of the network, making adhesion irreversible. The development of irreversibly adhered microbes is a crucial phase in the evolution of sessile biofilms.

The formation of surface micro-communities is the subsequent stage. At the inception of biofilm formation, there is no competition for nutrients and space among the adhered microbes, resulting in a rapid proliferation of microbes and evenly distributed colonies on the inner surface of the network.

During the emergence, detachment, and spread of biofilms, the adhered microbial communities gradually complicate as microbial adhesion continues, and the number of adhered individuals increases. Throughout the growth period of the biofilm, the speed of microbial adhesion surpasses that of detachment, leading to a continual increase in the amount of adhered biomass. However, when the biofilm grows to a certain thickness, the diffusion of nutrients and oxygen to the inner layers is inhibited, causing the microbes deep within the biofilm to die due to a lack of nutrients and oxygen, which, in turn, triggers extensive detachment of the biofilm.

A mature biofilm is predominantly constituted of bacteria, fungi, protozoa, and large invertebrates. The microbial communities in the biofilm primarily encompass bacteria and eukaryotes [36,37,38]. A total of 90% of the bacterial community is composed of Proteobacteria, Actinobacteria, and Bacteroidetes phyla. The Proteobacteria mainly comprise α-Proteobacteria and β-Proteobacteria. In eukaryotes, amoebae prevail. Common opportunistic pathogens in biofilms include *Legionella pneumophila* and *Avian Tuberculosis*. For example, *Naegleria fowleri* is a free-living, trophic amoeba that is nearly ubiquitous in the environment and can be present in high numbers in warm waters [39]. The existence of amoebae provides a habitat for the proliferation of pathogens. Furthermore, the microbes in biofilms are primarily rod-shaped bacteria and cocci [23]. The biofilm on the inner wall of polyethylene (PE) pipes mainly consists of short rod-shaped bacteria, while that on the inner wall of ductile iron pipes primarily contains cocci [40].

Factors influencing the biofilm quality primarily include nutrient substrate, temperature, types of disinfectants, and the amount of disinfectant added [41].

Nutrient Substrate: Most of the bacteria in water supply networks are heterotrophic, necessitating the consumption of organic carbon for growth and reproduction [42]. Hence, organic carbon is the principal factor limiting their proliferation. Nevertheless, for autotrophic bacteria within the networks, the ammonia nitrogen and carbonates in the water have a significant impact on their proliferation. There is a variety of organic substances in the water, typically characterized by assimilable organic carbon (AOC) and biodegradable dissolved organic carbon (BDOC), that depict the capability of organic matter in water to promote microbial growth [43]. When AOC is 10–20 µg acetic acid carbon per litter, heterotrophic bacteria can hardly grow; the threshold value for BDOC is generally 0.2 mg/L. In water with relatively high AOC or BDOC, phosphorus will replace organic matter as the limiting factor for microbial growth.

Temperature: Water temperature is one of the crucial factors influencing microbial growth [43]. Above 15 °C, microbes exhibit higher activity. While it is impossible to change the temperature of water supply networks, understanding the influence of water temperature on various reaction processes within the networks can provide relevant references for the control of water quality.

Types of Disinfectants and Added Quantity: Currently, the China drinking water sanitation standards stipulate that the residual chlorine in the network terminus should not be less than 0.05 mg/L [44]. However, even with a residual chlorine as high as 3−5 mg/L, the formation of biofilms cannot be entirely inhibited. The type of disinfectant also significantly impacts the biofilm. For both free chlorine and chloramine disinfectants, the density of biofilms on the pipe wall increases as the disinfectant concentration decreases.

Both disinfectants have an effective bactericidal capacity [45]. However, in cast iron pipes, when the concentration of free chlorine is 3–4 mg/L, it is still challenging to control the growth of biofilms. Yet, when the concentration of chloramine exceeds 2 mg/L, it effectively reduces the amount of biofilm. This is because chloramines exhibit greater persistence, thereby prolonging their contact with microbes, facilitating penetration of the biofilm, and ultimately inactivating adhered microbes more effectively [46].

### 2.3. Interaction between Chemical Stability and Biological Stability of Pipe Network Water Quality

Pipe scale, by adsorbing various organic matter present in the water body (such as proteins and polysaccharides), fosters an amenable environment for biofilm formation. The recesses and tubercles in pipe scale accumulate nutrients and safeguard microbes against hydraulic scouring and the harmful effects of disinfectants. Furthermore, the literature indicates that pipe scale accelerates the decay of disinfectants [47,48,49], which is beneficial for the growth and reproduction of microbes in biofilms. Therefore, to achieve effective disinfection of biofilms, it is imperative first to control pipe corrosion.

Microbes influence pipe material corrosion via metabolic actions, chiefly manifesting in the following ways [50]: (1) affecting anodic or cathodic reactions of electrochemical corrosion; (2) causing pitting corrosion on the metal surface; (3) the corrosive effect of acidic substances produced during microbial metabolism on the metal surface; and (4) promoting anaerobic corrosion by causing local anaerobic conditions. For instance, the microbes mainly associated with biocorrosion of iron pipes include iron oxidizing bacteria (IOB), iron reducing bacteria (IRB), sulfate oxidizing bacteria (SOB), and sulfate reducing bacteria (SRB).

IOB: These are bacteria capable of oxidizing Fe^2+^ to form Fe(OH)_3_ precipitation, where the energy released during this process satisfies their own metabolic needs. This oxidation process primarily involves three actions [51]: (1) The production of enzymes within bacterial cells, which directly promote the oxidation of ferrous iron to ferric iron, resulting in the formation of Fe(OH)_3_ precipitation that is excreted. This type of IOB, known as Type I IOB, mainly includes chemolithoautotrophic iron bacteria and facultative heterotrophic iron bacteria. Among them, chemolithoautotrophic iron bacteria include *Gallionella ferruginea* and *Leptothrix ochracea*. Facultative iron bacteria mainly include *Leptothrix discophora* and *Crenothrix polyspora*; (2) The exopolysaccharides (EPS) secreted by bacteria catalyze the oxidation of ferrous iron to ferric iron, forming Fe(OH)_3_ precipitation. This type of IOB is known as Type II IOB; (3) When iron is chelated by organic matter, heterotrophic iron bacteria can utilize the organic part of the chelate, and the released iron forms Fe(OH)_3_ precipitation either by catalyzed oxidation or directly. This type of IOB is referred to as heteroxygen IOB.

Autotrophic iron oxidizing bacteria, through their inherent metabolic processes, convert ferrous iron into Fe(OH)_3_, a biological oxidation rate far exceeding that of pure chemical oxidation [52]. Concurrently, the resulting Fe(OH)_3_ precipitation adheres to the pipe wall surface, creating small anodic points on the metal surface. These combine with the extensive cathodic region formed by high concentration oxygen to create a galvanic cell, leading to local pitting corrosion. Studies have demonstrated that Fe(OH)_3_ demonstrates anionic selectivity only at medium-to-low pH, so the precipitation of Fe(OH)_3_ allows anions to enter the metal surface, thereby preventing metal ions from overflowing from the metal surface. This results in an accumulation of chemical concentration, further promoting the occurrence of pitting corrosion. The dense deposits of Fe(OH)_3_ provide a living environment for anaerobes such as SRB. When SRB and IOB coexist, the iron corrosion rate is over 300 times that in EC conditions.

SRB, or sulfate-reducing bacteria, are a type of obligate anaerobe that utilizes organic matter as an electron donor and SO_4_^2–^ as a terminal electron acceptor, obtaining energy by reducing SO_4_^2–^ to S^2–^ (14).

(14) $${4\mathrm{Fe}+\mathrm{SO}}_{4}{}^{2-}{+\;4H}_{2}O\to \mathrm{FeS}+3\mathrm{Fe}(\mathrm{OH}{)}_{2}{+\;2\mathrm{OH}}^{-}$$

They are primarily from two bacterial genera: *Desulfovibrio* and *Desulgotomaculum*. In the water supply network, SRB are primarily *Desulfovibrio*, and they reside within the pipe scale.

At present, the mechanism of SRB promoting corrosion is mainly based on the cathodic depolarization theory. The hydrogenase possessed by SRB can remove hydrogen atoms from the cathode area, promoting the cathodic depolarization reaction in the corrosion process [53]. Moreover, mechanisms by which SRB promote metal corrosion also include concentration cell action, local cell action, metabolic product corrosion, deposit acid corrosion, and anode area fixed corrosion.

SOB, or sulfate oxidizing bacteria, are a type of aerobic bacteria capable of oxidizing sulfur, thiosulfate, and sulfite to produce sulfuric acid. They primarily include *Thiobacillus Thloparus*, *Thiobacillus Ferrooxidans*, and *Thiobacillus thiooxidans*. Under anaerobic conditions, SOB require nitrate (NO_3_^–^) as an electron acceptor (15) [54,55].

(15) $${5\mathrm{HS}}^{-}{+\;8\mathrm{NO}}_{3}{}^{-}{+\;3H}^{+}\to {\;5\mathrm{SO}}_{4}{{}^{2}}^{-}{+\;4N}_{2}{\;+4H}_{2}O$$

Iron-reducing bacteria (IRB) constitute a class of bacteria capable of reducing trivalent iron into divalent iron. Studies indicate that IRB can convert trivalent iron in Fe(OH)_3_ and Fe_2_O_3_ into divalent iron, subsequently amalgamating to form Fe_3_O_4_ (16) [56,57].

(16) $${\mathrm{Fe}}^{2+}{+\;2\mathrm{Fe}}^{3+}{+\;8\mathrm{OH}}^{-}\to {\;\mathrm{Fe}}_{3}O_{4}{+\;4H}_{2}O$$

Nitrate-reducing bacteria (NRB) under neutral conditions are able to use nitrate (NO_3_^–^) as an electron acceptor for the anaerobic oxidation of divalent iron [58,59]. In neutral conditions, the redox potential of NO_3_^–^/N_2_ is considerably higher than that of Fe^3+^/Fe^2+^; thus, during the NO_3_^–^ reduction process, it can accept electrons supplied by Fe^2+^, generating adequate energy for microbial growth and reproduction (17). Additionally, in the presence of molecular oxygen, NRB are similarly capable of oxidizing divalent iron [60,61,62].

(17) $${10\mathrm{Fe}}^{2+}{+\;2\mathrm{NO}}_{3}{}^{-}{+\;24H}_{2}O\to \;10\mathrm{Fe}{\left(\mathrm{OH}\right)}_{3}{+\;N}_{2}{+\;18H}^{+}$$

Due to the oxidizing action of IOB, oxygen undergoes reduction reactions in the cathodic region, while iron experiences oxidation reactions in the anodic region. The surface of the water supply pipes is subject to biological corrosion of Fe/Fe^2+^/Fe^3+^, resulting in the formation of Fe(OH)_3_ precipitation. When dissolved oxygen is present in the pipe network, the corrosion rate accelerates, leading to the generation of FeOOH. Experimental research has indicated that under the influence of IOB, amorphous iron hydroxide is initially formed in weakly acidic and alkaline solutions, swiftly transforming into α-FeOOH and Fe_2_O_3_ [20]. IRB can reduce the trivalent iron in Fe(OH)_3_ and Fe_2_O_3_ into divalent iron, further combining to produce Fe_3_O_4_. The iron cycle under microbial influence depends on the ability of IOB and IRB to obtain energy from the redox process of iron. Under the influence of SRB, iron oxides can be transformed into iron sulfide compounds. Jeffrey et al. observe that spherical iron hydrate compounds (rust layer) on the steel surface immersed in a tropical marine environment can gradually transform into flaky hexagonal iron sulfide compounds under microbial action [63]. They also noticed microbial attachment on the surface of spherical iron oxides, speculating that SRB may be involved in this reaction process.

## 3. Generation and Removal of Pipe Network Disinfection By-Products

In order to maintain the stability of the microbiota within the piping network, it is necessary to retain a certain disinfectant concentration at the end of the network. For instance, when utilizing free chlorine and monochloramine as disinfectants, the endpoint concentration shall not fall below 0.05 mg/L. Hence, the concentration of disinfectants and the production of disinfection by-products within the network warrant careful attention. Corrosive by-products from the piping network significantly impact the decay of disinfectants and the formation of disinfection by-products, operating through mechanisms such as scale-catalysis and scale-oxidation [15,18,47,48,62,64,65,66,67,68,69].

Corrosive by-products from the piping network can facilitate the decay of disinfectants. Multiple articles investigated the effect of copper corrosion by-products (such as CuO, Cu_2_O, and Cu^2+^) on disinfectant decay, discovering that these copper by-products can catalyze the accelerated decay of free chlorine [15,70,71]. Liu and his team find that CuO and NiO in the piping network can promote the disproportionation reaction of HOBr, thereby catalyzing the formation of BrO_3_^−^ [68]. Zhang and Andrews report that Cu^2+^ can catalyze the conversion of monochloramine into dichloramine in water [49]. Lin and associates discover that the corrosion by-product of lead pipes (PbO_2_) can oxidize chloramine, thereby releasing Pb^2+^ into the water body [72]. Berbey et al. find that in the presence of Br^−^, divalent manganese could be oxidized to MnO_2_ by free chlorine, leading to disinfectant decay [73]. Onset of nitrification is found also to accelerate chloramine decay [74]. Nitrification is a two-step microbiological process where ammonia is first oxidized to nitrite by ammonia-oxidizing bacteria (AOB), and finally, nitrite is oxidized to nitrate by nitrite-oxidizing bacteria (NOB) [75]. Recent studies have reported that in addition to pH and nitrite, soluble microbial products (SMPs) resulting from microbial activities significantly expedite chloramine decay by catalyzing auto-decomposition and nitrite oxidation processes in chlorinated waters [74,76,77].

Corrosive by-products from the piping network can also influence the formation of disinfection by-products [78]. Li et al. and Azeem et al. indicate that the presence of copper corrosion by-products (such as CuO, Cu_2_O, and Cu^2+^) can promote the formation of trihalomethanes (THMs) [15,79]. Zhang and Andrews reported that Cu^2+^ can enhance the formation of halogenated acetic acids (HAAs) and nitrosodimethylanilines (NDMAs) [48,49]. Liu et al. reveal that the presence of CuO can simultaneously encourage the formation of BrO_3_^−^ and THMs (Table 1) [80]. Gallard et al. find that MnO_2_ can oxidize I^−^ in the water body into HOI/I_2_, which then reacts with organic matter in the water to form more toxic iodinated disinfection by-products [81]. Lin and his team discover that PbO_2_ can also oxidize I^−^ in the water body into HOI/I_2_, subsequently transforming into iodoform (CHI_3_). Chun et al. report that trichloronitromethane (TCNM) in the Fe^2+^/FeOOH and Fe^2+^/Fe_3_O_4_ systems is degraded through reduction; trichloroacetonitrile (TCAN), 1,1,1-trichloropropanone (1,1,1-TCP), and trichloroacetaldyde (TCAh) are degraded through hydrolysis and reduction; while trichloromethane (TCM) and trichloroacetic acid (TCAA) cannot be degraded [82].

The corrosive by-products of the piping network facilitate the decay of disinfectants, severely jeopardizing the stability of water quality, and concomitantly escalating the production of disinfection by-products, thus amplifying the health risks associated with drinking water quality. Given the ubiquity of natural organic matter (NOM) in drinking water sources, and its resistance to conventional water treatment processes, it reacts with residual chlorine to generate disinfection by-products [83]. Moreover, metabolites from the microbial life within biofilms, secreted extracellular polymeric substances (EPS), and the organisms themselves increase the organic content of the water. When residual chlorine is present, these organic materials become precursors to disinfection by-products (DBPs), spawning an increased quantity or toxicity of DBPs. The interplay between residual chlorine and biofilms on pipe walls, leading to the formation of DBPs, is a concern that cannot be overlooked. Research by Wang et al. reveals a positive correlation between the formation of DBPs and a logarithmic reduction in the number of *Escherichia coli*, implying that bacterial lysis can release DBP precursors [84]. Biofilms dominated by *Pseudomonas aeruginosa*, along with planktonic bacteria of the same species, both serve as precursors to DBPs, with planktonic bacteria generating seven to eleven times the quantity of trihalomethanes (THMs) compared to biofilms. Whether parts of the biofilm enter the flowing water body or remain adhered to the pipe walls, they interact with residual chlorine, becoming the principal precursors to a significant increase in pipe network disinfection by-products, forming DBPs with carcinogenic, mutagenic, and teratogenic effects. Consequently, changing disinfectants becomes a key strategy for inhibiting the formation of disinfection by-products within the pipe network. Peroxymonosulfate (PMS) is a widely used industrial oxidant that also serves as a disinfectant, such as in swimming pool water or denture cleaning. PMS alone, similarly to hydrogen peroxide, is not as efficacious a disinfectant as active chlorine compounds [85]. If activated catalytically, thermally, or photolytically, however, the radicals formed are very energetic and effective oxidizing agents which may possess a potent disinfecting action [86,87]. More specifically, it is reported that PMS can be activated by transition metals, especially cobalt, or can be coupled with UV radiation [88,89]. During the disinfection process, PMS generates sulfate radicals (SO_4_•^−^) and hydroxyl radicals (HO•), which exhibit potent antimicrobial properties [88,90] (18–19). Poonsuk et al. find that utilizing PMS as a disinfectant can significantly reduce the load of Porcine Circovirus Type 2 in pig pens [91]. Anipsitakis et al. suggest that PMS has considerable potential in sterilizing reclaimed water facilities, such as against *Escherichia coli* [88]. However, PMS converts to SO_4_^2−^, which could exacerbate pipe network corrosion and result in iron release. Therefore, a comprehensive evaluation of iron release and disinfection by-product formation during the PMS disinfection process is warranted. In addition to suppressing the formation of disinfection by-products in the pipe network, water quality can also be purified at secondary or endpoint water supply. However, traditional water treatment processes (e.g., sedimentation, filtration, ozonation) and novel water treatment technologies (such as reverse osmosis) are ineffective at removing disinfection by-products [92,93]. Xiao et al. report that iodinated disinfection by-products (I-DBPs) can be rapidly degraded under ultraviolet irradiation [93]. Other studies have reported that chlorinated disinfection by-products (Cl-DBPs) and brominated disinfection by-products (Br-DBPs) can also be directly photodegraded by ultraviolet light or degraded by ultraviolet-based advanced oxidation technologies [94]. Therefore, ultraviolet degradation technology is currently the most promising technique for removing DBPs in secondary and endpoint water supplies [95,96].

(18) $${\mathrm{Co}}^{2+}{+\mathrm{HSO}}_{5}{}^{-}\to {\;\mathrm{Co}}^{2+}{+\mathrm{SO}}_{4}{}^{-}{+\mathrm{OH}}^{-}$$

(19) $${\mathrm{HSO}}_{5}{}^{-}+\mathrm{hv}\to {\;\mathrm{SO}}_{4}{}^{-}{+\mathrm{OH}}^{-}$$

## 4. Conclusions

The interconnectedness of factors contributing to disinfection by-product generation and the decay of disinfectants in drinking water distribution systems necessitates a multifaceted approach to ensuring water safety. Disinfectants and their decay mechanisms, corrosive by-products of the piping network, biofilm interactions, and disinfection by-product formation and removal techniques all play vital roles in maintaining the stability of drinking water. Therefore, it is essential to reasonably control the type and concentration of disinfectants in order to regulate chemical and microbial stability within the pipeline network, thereby reducing the formation of disinfection by-products. It is evident that using PMS as a disinfectant and the application of ultraviolet degradation technology for DBP removal show promise in addressing these challenges. However, more comprehensive studies are required to assess their long-term implications on water quality and public health. Striking the right balance between disinfection efficacy and minimizing harmful by-products remains an ongoing challenge, making this a significant focus for future research in drinking water safety.

## Figures and Tables

**Table 1:** The indices of disinfectants in drinking water [80].

Name of Disinfectant	Contact Time with Water (min)	Treated Water Limit (mg/L)	Ex-Treated Water Allowance (mg/L)	Water Allowance at the End of Pipe Network (mg/L)
Free chlorine	≥30	4	≥0.3	≥0.05
Monochloramine	≥120	3	≥0.5	≥0.05
Chlorine dioxide	≥30	0.8	≥0.1	≥0.02
ozone	≥12	0.3		≥0.02

## Data Availability

Not applicable.
